# Investigation of polygenic risk scores and subphenotypes in social anxiety disorder

**DOI:** 10.1038/s41398-026-04155-7

**Published:** 2026-07-11

**Authors:** Lisa Sindermann, Angelina Röhrig, Friederike S. David, Börge Schmidt, Katharina Domschke, Verena Nieratschker, Udo Dannlowski, Elisabeth J. Leehr, Eric Leibing, Markus M. Nöthen, Franziska Geiser, Johannes Schumacher, Carlo Maj, Rupert Conrad, Andreas J. Forstner

**Affiliations:** 1 Institute of Human Genetics, University of Bonn, School of Medicine & University Hospital Bonn, Bonn, Germany; 2 Department of Psychiatry and Psychotherapy, University Hospital Bonn, Bonn, Germany; 3 Department of Psychiatry and Psychotherapy, University of Marburg, Marburg, Germany; 4 Institute for Medical Informatics, University Hospital of Essen, Essen, Germany; 5 Department of Psychiatry and Psychotherapy, Medical Center – University of Freiburg, Faculty of Medicine, University of Freiburg, Freiburg, Germany; 6 German Center for Mental Health (DZPG), Partner Site Berlin/Potsdam, Berlin, Germany; 7 Department of Psychiatry and Psychotherapy, University of Tübingen, Tübingen, Germany; 8 German Center for Mental Health (DZPG), Partner Site Tübingen, Tübingen, Germany; 9 University of Münster, Institute for Translational Psychiatry, Münster, Germany; 10 Department of Psychiatry, Medical School and University Medical Center OWL, Protestant Hospital of the Bethel Foundation, Bielefeld University, Bielefeld, Germany; 11 German Center for Mental Health (DZPG), Site Jena Magdeburg Halle, Germany; 12 Center for Intervention and Research on Adaptive and Maladaptive Brain Circuits Underlying Mental Health (C-I-R-C), Site Jena Magdeburg Halle, Germany; 13 Clinic of Psychosomatic Medicine and Psychotherapy, University Medical Center Göttingen, Göttingen, Germany; 14 Department of Psychosomatic Medicine and Psychotherapy, University Hospital Bonn, University of Bonn, Bonn, Germany; 15 Centre for Human Genetics, University of Marburg, Marburg, Germany; 16 Department of Psychosomatic Medicine and Psychotherapy, University of Münster, Münster, Germany; 17 Institute of Neuroscience and Medicine (INM-1), Research Center Jülich, Jülich, Germany

**Keywords:** Genomics, Psychiatric disorders

## Abstract

Social anxiety disorder (SAD) is a common anxiety disorder (ANX) with moderate heritability that often co-occurs with other mental disorders. Until now, sample sizes in genetic analyses of SAD have been limited, so that the genetic basis of SAD and its subphenotypes is still largely unknown. In a large cohort comprising *n* = 1,194 SAD patients derived from five German cohorts and *n* = 3,409 controls from the Heinz Nixdorf Recall Study, we computed polygenic risk scores (PRS) at six *p*-thresholds using PRSice-2 based on large-scale genome-wide association studies for depression, major depressive disorder (MDD), ANX, schizophrenia (SCZ), bipolar disorder (BD), attention-deficit/hyperactivity disorder (ADHD), anorexia nervosa (AN), autism spectrum disorder (ASD), and alcohol dependence (AD). We used general linear models to examine the association between the PRS and SAD status. In SAD subsamples, we investigated whether the PRS are associated with SAD subphenotypes (i.e., SAD severity, current depressive symptoms, comorbid MDD) using correlation analyses and a general linear model. Results were corrected for multiple testing. The SAD status was significantly associated with PRS for depression, MDD, ANX, SCZ, BD, AN, and ASD (*p*_*BH*_-adjusted<0.05), but not with PRS for ADHD and AD. In SAD subsamples, the subphenotype analyses revealed no significant associations after correction for multiple testing (*p*_*BH*_-adjusted>0.05). Our results support that SAD seems to be genetically highly overlapping with other mental disorders, which might underline a common psychopathological factor. No significant association was found with SAD severity, current depressive symptoms or comorbid MDD. A better understanding of the genetic architecture of SAD may help to develop new diagnostic and treatment approaches.

## Introduction

Social anxiety disorder (SAD) represents one of the most common anxiety disorders (ANX) worldwide, with a 12-month prevalence of up to 7.1% [1]. SAD is characterized by an intense and persistent fear in social situations due to the expectation of being negatively evaluated, which usually results in avoidance behaviour [2]. As such, SAD causes increased socioeconomic burden and reduced quality of life [3, 4]. SAD often co-occurs with other mental disorders, such as comorbid major depressive disorder (SAD + MDD) [5, 6], which is linked to increased disease severity and disability [7]. Depressive symptoms often follow an SAD diagnosis [8] and patients with SAD frequently attempt to self-medicate with alcohol [9, 10]. Additionally, higher social anxiety symptoms were commonly present in patients with anorexia nervosa (AN) [11, 12] as well as patients with autism spectrum disorder (ASD) (for review see: [13]. Those comorbidities and overlapping symptoms underline the diagnostic difficulty of classifying a single disorder or comorbidity with the standard symptom-based classification system [14].

Furthermore, the potential diagnostic subtypes of SAD that go beyond the current classifications are intensively debated; some assume that SAD should be broken down into more dimensional subtypes with evidence supporting the use of dimensional measures (e.g., clinical severity, depressive symptom comorbidity) [15]. Biologically, potential SAD subtypes, such as SAD with varying degrees of severity, SAD with current depressive symptoms or SAD + MDD, remain insufficiently confirmed. Yet, being able to identify biologically relevant SAD subtypes would have important clinical implications: For example, treatment recommendations may differ and could be specifically adjusted for specific SAD subtypes in terms of personalized medicine. Further, identifying potential biomarkers could help to predict disease development or even prevent disease onset [16].

SAD seems to be influenced by genetic factors, with heritability estimates between 13% and 76% [17]. The relative risk for (generalized) SAD was found to be around tenfold greater in first-degree relatives of patients with SAD compared to controls without SAD [18]. Until now, SAD has usually been subsumed as a part of ANX in genetic studies (e.g., [19, 20]). The genome-wide association study (GWAS) on ANX by Purves et al. [20] included 25,453 patients with five core diagnoses of ANX (i.e., generalized anxiety disorder, SAD, panic disorder, agoraphobia, and specific phobia) and 58,113 controls, and identified five genome-wide significant loci [20]. In addition, genetic correlation analyses showed significant positive correlations between ANX and several other mental disorders such as schizophrenia (SCZ), bipolar disorder (BD), and attention-deficit/hyperactivity disorder (ADHD), indicating a degree of shared genetic risk [20–22]. Moreover, Romero and colleagues [23] reported a genetic overlap between ANX derived from the Million Veteran Program and depression as well as alcohol use disorder. However, we still lack understanding of the specific genetic links between mental disorders and SAD. Notably, the results of GWAS for mental disorders can be used to calculate polygenic risk scores (PRS), which aggregate the genetic risk for a specific disorder at the individual level [24, 25].

Only few studies have investigated genetic factors in SAD. Among others, the present authors investigated 24 single-nucleotide polymorphisms (SNPs) associated with other mental disorders in 321 patients with SAD and 804 controls [26]. In this study, none of the SNPs were associated with SAD after correction for multiple testing. Stein and colleagues [27] conducted a GWAS investigating soldiers answering a self-administered questionnaire to cover social anxiety symptoms. They found one genome-wide significant SNP (rs708012) to be linked to social anxiety symptoms [27]. However, compared to the investigation described in the present manuscript, previous genetic studies used a broader definition of SAD such as social anxiety symptoms [27], and/or tended to have limited sample sizes [26, 28], resulting in a reduced power to detect small effects [29]. Thus, the genetic architecture of SAD is not fully understood. Particularly, limited knowledge exists on the genetic contribution to SAD subphenotypes (e.g., SAD severity, SAD with current depressive symptoms, SAD + MDD).

To overcome these limitations associated with broad definitions of SAD, the lack of genetic knowledge around SAD, and limited sample sizes in genetic studies on SAD, we aimed to investigate the genetic architecture of SAD and its subphenotypes in a large sample comprising *n* = 1,194 patients with SAD and *n* = 3,409 controls. We aimed to elucidate the relationship between the genetic risk for a broader definition of depressive symptoms (depression), MDD, ANX, SCZ, BD, ADHD, AN, ASD, and alcohol dependence (AD) with (i) SAD case-control status as well as (ii) SAD subphenotypes (SAD severity, SAD with current depressive symptoms, SAD + MDD). Based on the literature, we hypothesized to find an association of PRS for those other mental disorders with SAD status (H1) and SAD subphenotypes (H2).

## Methods

### Subjects

The current cross-sectional study comprised phenotypical and genetic data of patients diagnosed with SAD. They derived from studies at five sites: Bonn [30], Göttingen [31], Münster (detailed study description will be published elsewhere), Münster-Freiburg [32, 33], and Tübingen [34–36]. The controls consisted of a population-based cohort derived from the Heinz Nixdorf Recall Study [37, 38].

### Ethics approval and consent to participate

All participants provided written informed consent before study participation. The study was approved by the institutional ethics committee of the Medical Faculty of Bonn (reference number: 222/12. All methods were performed in accordance with the relevant guidelines and regulations.

SAD patients were recruited from different sources (e.g., flyer, clinical settings) and could be either in-patients or out-patients. All individuals with SAD fulfilled the criteria for the diagnosis SAD as determined by structured clinical interviews (see Materials and clinical Assessment). The detailed inclusion and exclusion criteria of the sites can be found in the study references mentioned above. Briefly, the general inclusion criteria for SAD patients were (i) age 18 years and older, (ii) a lifetime diagnosis of SAD. SAD patients from Göttingen also had to have (i) a primary diagnosis of SAD and (ii) a Liebowitz Social Anxiety Scale (LSAS) [39] above 30 [40]. General exclusion criterion was inadequate German language skills for completing the study instruments.

The controls (aged between 45 to 75 years) were randomly selected from mandatory residence lists in Essen, Bochum, and Mülheim. According to Schmermund and colleagues (2002), they had no severe mental disorders. A presence of SAD, however, cannot be completely excluded.

### Materials and clinical assessment

In general, patients with SAD underwent a clinical interview, completed a psychological battery, and provided blood or saliva samples for genetic analyses. Controls underwent questions about their lifestyle, provided blood samples, and gave cardiovascular measurements [37].

Regarding the clinical interview, the diagnosis of a lifetime SAD (and SAD + MDD) was assessed by trained interviewers using the German version of the Structured Clinical Interview for DSM-IV-TR Axis I Disorders (SCID-I) [41]. The controls from the Heinz Nixdorf Recall Study did not undergo such clinical testing (see Limitations).

Regarding the psychological battery, not all questionnaires were available at all sites (compare Table 1 for numbers after quality control). In addition, no psychometric data were available for the controls (cf. Limitations). In SAD, the current severity of social anxiety symptoms (over the past week) was assessed using the German version of the self-reported Social Phobia Inventory (SPIN) [42] only at the Bonn site (*n* = 658). Hereby, the SPIN comprises 17 items answered on a five-point Likert scale from zero (not at all) to four (extremely), with a total sum score ranking from 0 to 68 [42].

**Table 1:** Sociodemographic and clinical data.

	SAD (*n* = 1,194)					Controls (*n* = 3,409)	Test-statistics	*p*-value	Effect sizes
	Münster (*n* = 40)	Göttingen (*n* = 258)	Bonn (*n* = 724)	Münster-Freiburg (*n* = 107)	Tübingen (*n* = 65)	Heinz Nixdorf Recall			
SAD + MDD%, *n* = 1112	80.00%	24.27%	74.02%	18.69%	61.54%	-	256.59^a^	2.5e-54	0.480 ^d^
BDI-I, *n* = 1,021	n.a.	13.79(8.78)	19.80(11.01)	13.47(8.82)	n.a.	-	41.82^b^	3.5e-18	0.076^e^
BDI-II, *n* = 105	13.88(9.85)	n.a.	n.a.	n.a.	14.18(9.41)	-	-0.16^c^	0.87	-
SPIN, *n* = 658	n.a.	n.a.	40.60(11.62)	n.a.	n.a.	-	-	-	-
LSAS, *n* = 363	66.55(22.43)	71.69(21.80)	n.a.	n.a.	71.52(27.19)	-	0.89^b^	0.41	-
Sex, *n* = 4603 (male/ female%)	49.60%/ 50.40%					43.13%/56.87%	14.84^a^	1.2e-04	0.057 ^d^
Age, *n* = 4589	37.02(13.69)					60.13(7.84)	70.84^c^	<0.001	9.705 ^f^

*Notes.* Depending on the scale level, we present different statistical values and different measures for effect sizes. ^a^ *X²*-value, ^b^ *F*-value, ^c^ *t*-value, ^d^ Cramer’s *V*, ^e^ partial eta², ^f^ Cohen’s *d*. *SAD* social anxiety disorder, *SAD* + MDD%, percentage of social anxiety disorder with comorbid major depressive disorder; *BDI-I* Beck Depression Inventory version I, *BDI-II* Beck Depression Inventory version II, *SPIN* Social Phobia Inventory, *LSAS* Liebowitz Social Anxiety Scale, n.a., not available.

Additionally, the current severity of social anxiety symptoms (over the past week) was assessed using the German version of the self-reported Liebowitz Social Anxiety Scale (LSAS) [39] at the Münster, Göttingen, and Tübingen sites (*n* = 363). The LSAS consists of 24 items addressing fear/anxiety and avoidance that can be answered on a scale from zero (none or never) to three (severe or usually), with a sum score ranking from 0 to 144 [39].

In SAD individuals, the severity of current depressive symptoms (over the past week) was investigated using the German version of the Beck Depression Inventory (BDI-I) [43] at the Bonn, Göttingen, and Münster-Freiburg sites (*n* = 1,021). The BDI-I consists of 21 items on a scale from zero (symptom absent) to three (severe symptoms), and sum scores are between 0 and 63 [43].

The Beck Depression Inventory Revision (BDI-II) [44] was used at the Münster and Tübingen sites (*n* = 105) measuring depressive symptoms over the past two weeks. The BDI-II represents the revised version of the BDI-I replacing four items. The questionnaire also consists of 21 items on a scale from zero (symptom absent) to three (severe symptoms), and sum scores are between 0 and 63 [44].

### Genotyping, quality control, and imputation

The participants’ DNA was extracted from blood or saliva samples using standard methods. Genome-wide genotyping was performed using the Infinium Global Screening Array (GSA, Illumina, San Diego, CA, USA).

Genome-wide genotyping, quality control, and imputation were conducted in a larger sample, of which the present study sample constitutes a subset. Genotyping quality control (QC) was performed according to the Rapid Imputation and Computational Pipeline (RICOPILI) [45]. At the sample level and at the SNP level, the following criteria had to be fulfilled: Samples were excluded if they had a call rate ≤98% or discordant information between the phenotypic and genotypic sex. Variants were excluded if they had a call rate ≤98% or failed the Hardy-Weinberg equilibrium (HWE) test (*p* < 1e-10 for patients with SAD or *p* < 1e-6 for controls). A relatedness check was performed using the tool KING v.2.1 [46] excluding individuals with a kinship coefficient >0.0442 (indicating a third-degree relationship or closer genetic relationship). In order to investigate the population structure, individual genotypes were projected to the principle components space by running a principal component analysis (PCA). We used individual data from 1000 Genomes Project Phase 3 [47] assigning individuals to one of the five superpopulations (i.e., Africa, East Asia, Europe, South Asia, and America). Individuals were assigned according to the geometric distance with respect to the centroid of the respective superpopulation by using PLINK 1.9 [48] and custom R scripts (adapted from https://github.com/privefl/paper-ancestry-matching/tree/master/code). Individuals that clustered with a non-European population were excluded as well as outliers ( ± 6 SD) of the means of the first 10 principal components.

The imputation was run on the shared subsets of variants (with non-solvable ambiguous variants removed during merging) including a total number of *n* = 437,470 SNPs. The imputation of genotype data was performed using Eagle v2 [49, 50] for phasing and Minimac4 (https://genome.sph.umich.edu/wiki/Minimac4) for imputation using the 1000 Genomes Project Phase 3 [47] reference panel. Only variants with a Minimac imputation quality score (*R²*) ≥ 0.3 and minor allele frequencies (MAF) ≥ 1% were retained for the following analyses (*n* = 10,862,809 SNPs).

After quality control and imputation, a sample of *n* = 1,266 patients with SAD and *n* = 4,139 controls remained. Subsequently, the genetic data were merged with the phenotypic data resulting in the exclusion of *n* = 72 patients with SAD and *n* = 6 controls due to missing phenotypic data. Individuals with a known overlap between the GWAS samples (representing the base data for PRS calculation) and our sample (representing the target dataset) were also excluded (*n* = 724 of the controls). The final dataset comprised *n* = 4,603 individuals, including *n* = 1,194 patients with SAD and *n* = 3,409 controls.

### Polygenic risk scores

In the current cohort, nine PRS of mental disorders were calculated using PRSice-2 [51]. In those PRS, the individual genetic risk scores (proxy of cumulative risk) were derived from the linear additive combination of selected variants weighted according to the effect sizes of the following reference GWAS: Depression [52], MDD without 23andme data [53], ANX [20], SCZ [54], BD [55], ADHD [56], AN [57], ASD [58], and AD [59]. The sample sizes of these base GWASs are indicated in Table S1.

For QC, the listed base datasets were filtered for standard GWAS parameters (MAF > 1%, imputation information>80%). The standard pruning plus thresholding (P + T) approach was used as implemented in PRSice-2 [51] using six *p*-value thresholds ranking from genome-wide significance to the full model (P5e-08, P1e-06, P0.0001, P0.05, P0.5, P1) for all PRS except for the PRS of AD. For the PRS of AD only five *p*-thresholds (P5e-08, P0.0001, P0.05, P0.5, P1) could be calculated. A second PCA (cf. Genotyping, quality control, and imputation) within the final sample was performed. According to Figure S1, the first four principal components (PCs) of the PCA were used for further polygenic risk score analyses. Additionally, all PRS were z-standardized.

### Statistical analyses

For the descriptive and statistical analyses, we used IBM SPSS Statistics 29 software [60]. To investigate the sociodemographic and clinical data, we calculated *X²* tests to investigate group differences regarding sex and independent *t*-tests (two-sided) regarding age. We investigated SAD group differences depending on the sites (Münster, Göttingen, Bonn, Münster-Freiburg, Tübingen) by running a *X²* test for SAD + MDD, independent *t*-test (two-sided) regarding the BDI-II, and analysis of variance for the BDI-I and LSAS. For the SPIN we are only able to show descriptive statistics because only one site (Bonn) used this questionnaire. Additionally, we checked for group differences in SAD vs. SAD + MDD by running *X²* tests and independent *t*-tests (two-sided) (cf. Table S2).

The analysis strategy was twofold: First, we investigated the link between the nine PRS (depression, MDD, ANX, SCZ, BD, AN, ADHD, ASD, AD) at each of the *p*-thresholds and the SAD case-control status by using general linear models controlling for sex and the first four PCs. We corrected for multiple testing (53 tests) using the false discovery rate adjusted *p*-value according to Benjamini-Hochberg and claimed significance at *p*_*BH*_-adjusted<0.05. When the assumption of homogeneity of variances using Leven’s test was violated (*p* < 0.05) (cf. Table S3), we additionally run Welch’s Analysis of Variance and claimed significance at *p*_*BH*_-adjusted<0.05 (14 tests).

Second, we exploratory investigated the link between the significant PRS and potential SAD subphenotypes (SAD severity, SAD with current depressive symptoms, SAD + MDD). Psychometric data were available for some of the patients with SAD (SPIN: *n* = 658, LSAS: *n* = 363, BDI-I: *n* = 1,021, BDI-II: *n* = 105). Thus, calculations were performed in these SAD subsamples based on data availability (cf. Materials and clinical assessment). Thus, we calculated partial correlations controlling for sex, the first four PCs, and age to explore the association with current SAD severity (measured with the SPIN and LSAS) as well as current depressive symptoms (measured with the BDI-I and BDI-II). In addition, we constructed a general linear model controlling for sex, the first four PCs, and age to elucidate potential differences between SAD and SAD + MDD. For those models, age was included due to its potential influence on the clinical data [61]. Results were corrected for multiple testing (32 tests) with the Benjamini-Hochberg method and claimed significant at *p*_*BH*_-adjusted<0.05.

No formal a priori sample size calculation was performed. With the available sample size (*n* = 4,603; *n* = 1,194 SAD cases and *n* = 3,409 controls), the main analyses using GLM had sufficient power ( > 80%) to detect small effect sizes (*partial eta²*≈0.02) at α = 0.05. Subgroup analyses using partial correlations (SPIN: *n* = 658, LSAS: *n* = 363, BDI-I: *n* = 1,021, BDI-II: *n* = 105) were adequately powered for moderate effects (*r²*≈0.06), with the smallest subset (BDI-II) being underpowered for small effects.

## Results

### Sociodemographic and clinical data

Table 1 displays the sociodemographic data of patients with SAD and controls as well as the clinical data in the SAD group. Controls were significantly older than patients with SAD (*t*(4587) = 70.78, *p* < 0.001, Cohen’s *d* = 9.705). Group differences in SAD vs. SAD + MDD can be found in Table S2.

### Polygenic risk scores of mental disorders and SAD case-control status

**Fig. 1:**
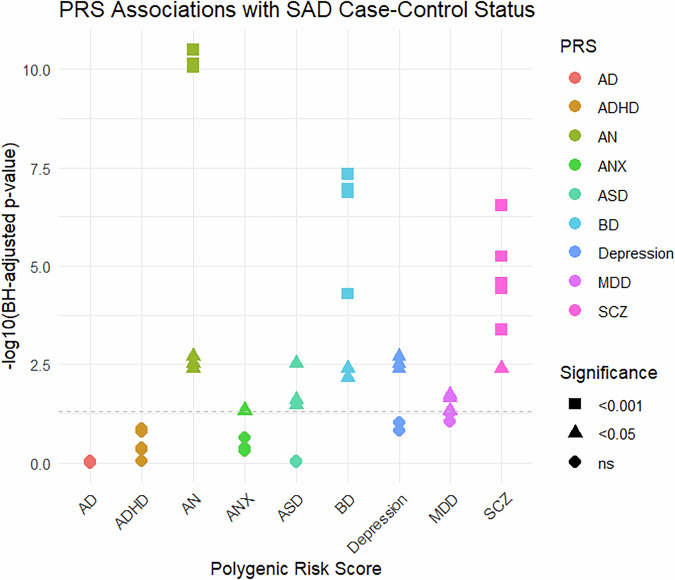
PRS associations with SAD case-control status. *Notes*. The figure illustrates the association between the SAD case-control status with the six different thresholds of the PRS (P5e-06, P1e-01, P0.0001, P0.05, P0.5, P1). Associations were tested using general linear models controlling for sex and the first four principal components. The x-axis displays the phenotypes and the y-axis represents the −log10 of Benjamini-Hochberg adjusted *p*-values, with higher values indicating stronger statistical significance. Significance is indicated above the dashed gray line, with *p* < 0.05 shown as triangles and *p* < 0.001 as squares. *Abbreviations*. BH Benjamini-Hochberg, PRS polygenic risk score, Depression, broader definition of depressive symptoms; MDD major depressive disorder, ANX anxiety disorder, SCZ schizophrenia, BD bipolar disorder, ADHD attention-deficit//hyperactivity disorder, AN anorexia nervosa, ASD autism spectrum disorder, AD alcohol dependence, SAD social anxiety disorder, ns not significant.

Table 2 and Fig. 1 illustrate the results of the main analysis testing group differences in SAD patients compared to controls regarding the nine PRS at each *p*-threshold. SAD patients compared to controls presented significantly increased PRS for depression and MDD at four thresholds (depression: P0.0001, P0.05, P0.5, P1; MDD: P5e-08, P1e-06, P0.0001, P1). They also presented increased PRS for ANX and ASD at three thresholds (P0.05, P0.5, P1) and showed increased PRS for SCZ, BD, and AN at all *p*-thresholds (P5e-08, P1e-06, P0.0001, P0.05, P0.5, P1) after correction for multiple testing using the Benjamini-Hochberg method. The PRS for ADHD and AD did not reach significance (all *p*_*BH*_-adjusted>0.05).

**Table 2:** Results of the PRS for SAD case-control status.

PRS	*p*-threshold	*F*-value	*p*-value	*p*_*BH*_-adjusted	*partial eta²*
Depression	P5e-08	2.563	1.09e-01	1.53e-01	-
Depression	P1e-06	3.404	6.51e-02	9.86e-02	-
Depression	P0.0001	9.793	1.76e-03	4.45e-03*	0.002
Depression	P0.05	10.472	1.22e-03	3.40e-03*	0.002
Depression	P0.5	11.625	6.56e-04	2.48e-03*	0.003
Depression	P1	11.361	7.56e-04	2.67e-03*	0.002
MDD	P5e-08	4.782	2.88e-02	4.77e-02*	0.001
MDD	P1e-06	6.892	8.69e-03	1.92e-02*	0.002
MDD	P0.0001	6.609	1.02e-02	2.16e-02*	0.001
MDD	P0.05	3.566	3.64e-02	5.85e-02	-
MDD	P0.5	1.037	5.96e-02	9.29e-02	-
MDD	P1	0.392	2.59e-02	4.74e-02*	0.001
ANX	P5e-08	0.989	3.20e-01	4.14e-01	-
ANX	P1e-06	1.841	1.75e-01	2.32e-01	-
ANX	P0.0001	0.624	4.30e-01	5.18e-01	-
ANX	P0.05	5.125	2.36e-02	4.47e-02*	0.001
ANX	P0.5	4.879	2.72e-02	4.66e-02*	0.001
ANX	P1	4.891	2.70e-02	4.78e-02*	0.001
SCZ	P5e-08	10.591	1.00e-03	3.12e-03*	0.002
SCZ	P1e-06	15.278	9.41e-05	4.16e-04*	0.003
SCZ	P0.0001	24.294	8.56e-07	5.67e-06*	0.005
SCZ	P0.05	30.343	3.82e-08	2.89e-07*	0.007
SCZ	P0.5	20.267	6.90e-06	3.66e-05*	0.004
SCZ	P1	20.953	4.83e-06	2.84e-05*	0.005
BD	P5e-08	9.84	1.71e-03	4.54e-03*	0.002
BD	P1e-06	8.87	2.91e-03	6.71e-03*	0.002
BD	P0.0001	19.43	1.07e-05	5.15e-05*	0.004
BD	P0.05	35.07	3.42e-09	4.53e-08*	0.008
BD	P0.5	32.91	1.02e-08	1.08e-07*	0.007
BD	P1	32.14	1.52e-08	1.34e-07*	0.007
ADHD	P5e-08	2.824	9.30e-02	1.37e-01	-
ADHD	P1e-06	0.024	8.76e-01	9.29e-01	-
ADHD	P0.0001	2.777	9.60e-02	1.38e-01	-
ADHD	P0.05	2.402	1.21e-01	1.64e-01	-
ADHD	P0.5	0.907	3.41e-01	4.30e-01	-
ADHD	P1	0.779	3.78e-01	4.66e-01	-
AN	P5e-08	10.043	2.00e-03	4.82e-03*	0.002
AN	P1e-06	11.934	5.56e-04	2.27e-03*	0.003
AN	P0.0001	10.447	1.00e-03	2.94e-03*	0.002
AN	P0.05	52.111	6.11e-13	3.24e-11*	0.011
AN	P0.5	49.134	2.74e-12	7.26e-11*	0.011
AN	P1	48.005	4.84e-12	8.55e-11*	0.010
ASD	P5e-08	0.031	8.61e-01	9.51e-01	-
ASD	P1e-06	0.015	9.02e-01	9.37e-01	-
ASD	P0.0001	0.089	7.66e-01	9.02e-01	-
ASD	P0.05	10.970	9.33e-04	3.09e-03*	0.002
ASD	P0.5	6.270	1.23e-02	2.51e-02*	0.001
ASD	P1	5.671	1.73e-02	3.39e-02*	0.001
AD	P5e-08	0.028	8.67e-01	9.38e-01	-
AD	P0.0001	0.037	8.48e-01	9.56e-01	-
AD	P0.05	0.046	8.31e-01	9.57e-01	-
AD	P0.5	1.3e-07	1.00e + 00	1.00e + 00	-
AD	P1	14.9e-04	9.82e-01	1.00e + 00	-

*Notes*. ^*^The asterisk denotes significance at *p*_*BH*_ < 0.05 corrected for multiple testing (53 tests) according to Benjamini-Hochberg.

*PRS* polygenic risk score, Depression, broader definition of depressive symptoms, *MDD* major depressive disorder, *ANX* anxiety disorder, *SCZ* schizophrenia, *BD* bipolar disorder, *ADHD* attention-deficit/hyperactivity disorder, *AN* anorexia nervosa, *ASD* autism spectrum disorder, *AD* alcohol dependence.

Descriptive statistics (mean, standard deviation) of the two groups and Levene’s test results can be found in Table S3. Levene’s test indicated unequal variances for 14 tests. The sensitivity analyses using Welch’s Analysis of Variance yielded similar conclusions (please compare Table S4).

### Polygenic risk scores and SAD subphenotypes

#### Polygenic risk scores and SAD severity

The association between the previously reported significant PRS and SAD severity did not reach significance after correcting for multiple testing using the Benjamini-Hochberg method (LSAS: smallest *p*_*BH*_-adjusted=0.302 for PRS for SCZ at P1; SPIN: smallest *p*_*BH*_-adjusted=0.838 for PRS for SCZ at P1; cf. Table S5).

#### Polygenic risk scores and SAD with current depressive symptoms

In SAD subsamples, we found no significant relationship between the previously reported significant PRS and the current depressive symptoms after correcting for multiple testing (BDI-I and BDI-II: all *p*_*BH*_-adjusted>0.05; cf. Table S5). However, we found a trend presenting a positive correlation between the BDI-I score and the PRS for depression at P0.5 (*p*_*BH*_-adjusted=0.06) (cf. Figure S2).

#### Polygenic risk scores and SAD + MDD

Table S6 presents the subgroup findings of testing group differences in SAD patients compared to SAD + MDD regarding the previously reported PRS. The PRS did not differ in SAD vs. SAD + MDD after correcting for multiple testing using the Benjamini-Hochberg method (smallest *p*_*BH*_-adjusted=0.104 for the PRS for AN at P5e-08).

## Discussion

In this study, we investigated the association of the PRS for depression, MDD, ANX, SCZ, BD, ADHD, AN, ASD, and AD with SAD case-control status and SAD subphenotypes (i.e., SAD severity [LSAS, SPIN], current depressive symptoms [BDI-I, BDI-II], and SAD + MDD). SAD case-control status was significantly linked at all *p*-thresholds with the PRS for SCZ, BD, and AN, at four thresholds with the PRS for depression and MDD, and at the three least stringent *p*-thresholds with the PRS for ANX and ASD. SAD and controls did not significantly differ in their PRS for ADHD and AD. In SAD alone, the investigated PRS were not significantly associated with SAD severity. However, we found a trend linking the current depressive symptoms in SAD to the PRS for depression. SAD and SAD + MDD did not differ regarding the PRS after correction for multiple testing.

We hypothesized we would find an association of PRS for mental disorders (depression, MDD, ANX, SCZ, BD, AN, ADHD, ASD, AD) with SAD status (H1) and SAD subphenotypes (H2). In line with H1, we found a significant association with PRS for several mental disorders suggesting a high degree of shared genetic risk of SAD with SCZ, BD, and AN as well depression, MDD, ANX, and ASD. These genetic findings hint to a shared psychopathological factor between SAD and a wide band of major mental disorders. This may also explain the overlapping clinical picture regarding shared symptoms [2, 62] as well as high comorbidity rates [63]. Nonetheless, the low effect sizes reported in our study should be taken into account. Our results thus provide evidence that previous findings of broad genetic overlaps between mental disorders such as SCZ and BD [23, 64] can be extended to SAD. Interestingly, previous studies reported a link between psychotic disorders and other types of ANX apart from SAD (i.e., panic disorder (PD) and generalized anxiety disorder (GAD)) [65–67], which supports the suggestion that several types of ANX, including SAD, show genetic overlaps with psychotic disorders. This might also contribute to the phenotypical overlap as SAD was found to be tenfold higher in patients with SCZ [68] and to co-occur in 13.5% of BD patients during their lifetime [69]. Our results provided evidence that SAD is not only genetically linked with psychotic disorders but also with AN; previously, AN was found to be genetically associated with PD and GAD as part of ANX [70, 71]. AN and SAD often co-occur [72, 73], and both disorders usually begin in the early to mid-teenage years [74, 75]. Notably, first-degree relatives of individuals with AN have a significantly higher prevalence of ANX (including SAD) [70]. Thus, a relationship might exist between diverse ANX (e.g., PD, GAD, SAD) and psychotic disorders and AN. Additionally, our results may partly explain the phenotypic overlap (e.g., social difficulties) in ASD and SAD [76]. Some monogenic forms of ASD exist (for a review see: [77]). Thus, future studies should investigate the genetic relationship between ASD and SAD not only at the level of common variants, but also at the rare variant level.

In contrast to our hypothesis H1, it is conceivable that the failure to find significant associations between SAD case-control status and the PRS for ADHD and AD may have arisen because of several reasons. We note that a stringent correction for multiple testing was applied. Thus, significant results should be even more robust. On the one hand, this finding may indicate that there is no genetic overlap between ADHD, AD, and SAD, which may be due to other biological mechanisms underlying the development of the disease. On the other hand, it is also possible that only very specific pathways overlap or that there might be only an overlap between specific subtypes of SAD, which should be investigated in future studies.

Regarding our second hypothesis H2, we expected to find an association between PRS of mental disorders and SAD severity, SAD with current depressive symptoms, and SAD + MDD. In SAD subsamples, SAD subphenotype analyses did not reach significance after correcting for multiple testing, which might be related to the limited sample size. Although, our results do not provide strong support that PRS for mental disorders (including ANX) are linked to SAD subphenotypes, future studies in larger samples should investigate the genetic architecture of these and further SAD subphenotypes before more definitive conclusions can be drawn.

### Limitations

The present study had several limitations. First, the included studies used different questionnaires to investigate SAD subphenotypes (severity of SAD, current depressive symptoms, SAD + MDD), which might have influenced the results of our secondary analyses by limiting the sample size. However, including different, but common questionnaires might also broaden the generalizability of the findings. As such, further characterized phenotypes and larger sample sizes are required to obtain more robust results. In addition, psychometric data were not available for the control group, which precluded their inclusion in the SAD subphenotype analyses. Future studies should consider including controls with obtained comparable psychometric measures to investigate gradient effects across groups (e.g., controls, SAD without comorbid MDD, SAD with comorbid MDD). Investigating SAD subphenotypes (severity of SAD, current depressive symptoms, SAD + MDD) is only one possible way to analyse SAD subgroups. The review by D’Avanzato and Dalrymple [15] describes several other ways of dimensional and categorical subtyping in SAD. Future studies should compare these different approaches to establish a consensus on which subtypes should be prioritized for investigation.

Second, we would like to note that the sample characteristics might also have partly influenced the obtained results. The comorbidity in our sample, especially, the increased rate of SAD + MDD may also have influenced the association between the PRS for depression and MDD with SAD case-control status. Future studies should investigate this finding in larger sample sizes of SAD patients without comorbidities. Furthermore, the HNR control individuals were not screened for the presence of SAD. This may have led to a reduced statistical power to detect associations. However, according to Moskvina and colleagues [78], this should not have had a major impact due to the relatively low lifetime prevalence of SAD, despite of being one of the most common ANX. Future studies are encouraged to investigate samples with screened controls in which SAD has been excluded.

Third, in this study we used PRSice-2 as a method to calculate the PRS. More recently, diverse methods for PRS calculation with different approaches have been published that perform best in different settings depending on several factors, such as genetic architectures of the traits and tuning parameters [79, 80]. To further address the proposed research questions, future studies should also use other PRS calculation methods.

## Conclusion

In the present study, we systematically investigated the association of PRS for nine mental disorders (depression, MDD, ANX, SCZ, BD, ADHD, AN, ASD, and AD) on SAD status and its subphenotypes (social anxiety severity, current depressive symptoms, and SAD + MDD). The results support the notion that SAD seems to be genetically highly overlapping with multiple mental disorders, including SCZ, BD, and AN, which might indicate a common psychopathological factor. We could not identify statistically significant associations of PRS with SAD’s subphenotypes. Overall, increasing our understanding of the genetic architecture of SAD may aid in refining diagnostic decisions and offering tailored treatment options.

## Supplementary information

Supplementary Materials

## Data Availability

The data and code used for the analyses in this study is available from the corresponding author upon reasonable request.
