# Supplementary material for: Investigation of polygenic risk scores and subphenotypes in social anxiety disorder
Source: Transl Psychiatry. 2026 Jul 11;16:356. doi: 10.1038/s41398-026-04155-7 (PMC13354785; doi:10.1038/s41398-026-04155-7)
Supplement: Supplementary file 1 — Supplementary Materials [file 41398_2026_4155_MOESM1_ESM.docx]

# Supplementary Materials

**Figure S1.** Scree plot of the first 10 principal components.

**Figure S2.** PRS for depression and current depressive symptoms in SAD.

**Table S1.** Information on the base GWASs.

**Table S2.** Sample characteristics of SAD vs. SAD+MDD.

**Table S3.** Descriptive statistics and Levene’s test results.

**Table S4.** Results of the Welch’s Analysis of Variance.

**Table S5.** Results of the association analyses between PRS and SAD subphenotypes.

**Table S6.** Results of the PRS comparing SAD and SAD+MDD.

## Figure S1

*Scree plot of the first 10 principal components.*


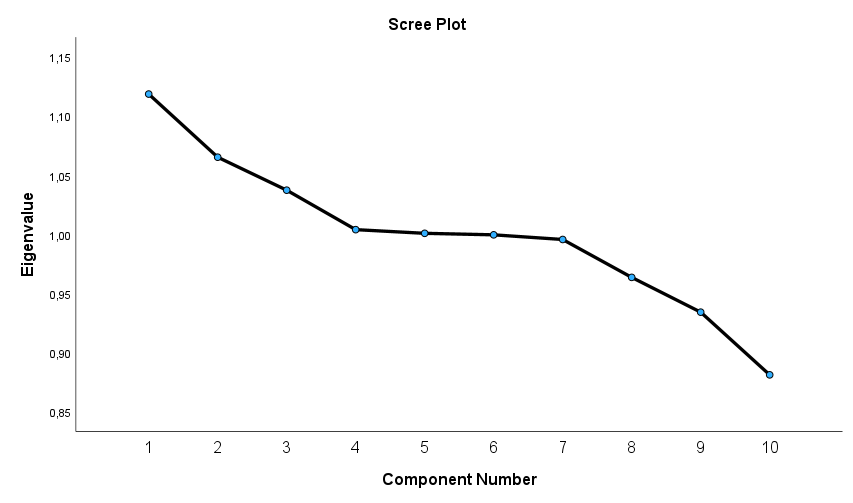


*Notes*. The first four principal components were used for further polygenic score analyses according to the scree plot above.

## Figure S2

*PRS for depression and current depressive symptoms in SAD.*

*
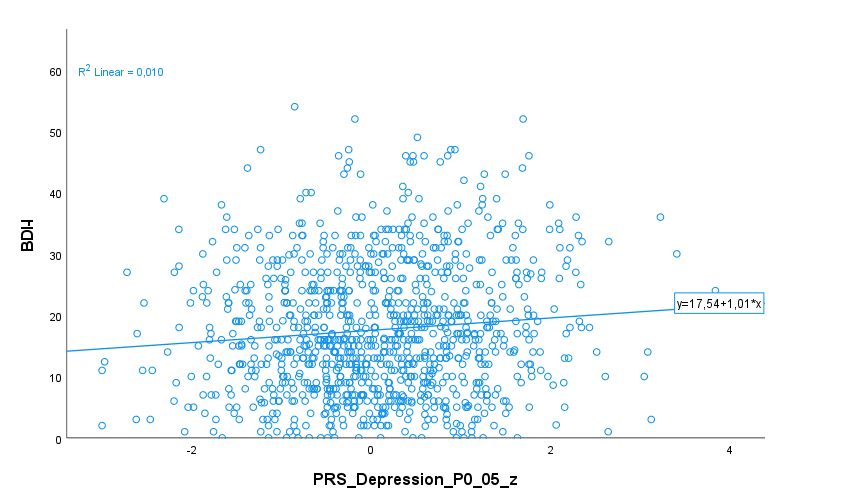
*

*Notes*. An increased trend was found between the PRS for depression P0.05 (on the x-axis) and the BDI-I score (on the y-axis) in patients with SAD (*p_BH_*-adjusted>0.05).

*Abbreviations.* BDI-I, Beck Depression Inventory version I; SAD, social anxiety disorder; PRS_Depression_P0_05_z, z-standardized polygenic risk score for depressive symptoms at the *p*-threshold 0.05.

## Table S1

*Information on the base GWASs.*

| First author (Year) | Phenotype | Sample size |
| --- | --- | --- |
| Howard et al. (2019) | Depression | Total 500,199, cases 170,756 cases, controls 329,443 ^a^ |
| Wray et al. (2018) | MDD | Total 173,005, cases 59,851, controls 113,154 ^a^ |
| Purves et al. (2020) | ANX | Total 83,544, cases 25,453, controls 58,113 |
| Pardiñas et al. (2018) | SCZ | Total 105,318, cases 40,675, controls 64,643 |
| Mullins et al. (2021) | BD | Total 51,710, cases 20,352, controls 31,358 |
| Demontis et al. (2019) | ADHD | Total 55,374, cases 20,182, controls 35,191 |
| Watson et al. (2019) | AN | Total 72,517, cases 16,992, controls 55,525 |
| Grove et al. (2019) | ASD | Total 46,350, cases 18,381, controls 27,969 |
| Walters et al. (2018) | AD | Total 52,848, cases 14,904, controls 37,944 |

*Notes.* The table displays general information on the base GWAS meta-analyses. *Abbreviations.* GWAS, genome-wide association study; PRS, polygenic risk score; Depression, broader definition of depressive symptoms; MDD, major depressive disorder; ANX, anxiety disorder; SCZ, schizophrenia; BD, bipolar disorder; ADHD, attention-deficit/hyperactivity disorder; AN, anorexia nervosa; ASD, autism spectrum disorder; AD, alcohol dependence.

## Table S2

*Sample characteristics of SAD vs. SAD+MDD.*

|  | SAD (*n*=472) | SAD+MDD (*n*=640) | Test-statistics | *p*-value | Effect sizes | |  |  |
| --- | --- | --- | --- | --- | --- | --- | --- | --- |
| Sex, *n*=1112 (male/ female%) | 45.76%/54.24% | 38.59%/61.41% | 5.75^a^ | 0.017 | 0.072^c^ | |  |  |
| Age, *n*=1112 | 36.34(13.34) | 37.81(14.02) | -1.77^b^ | 0.078 | - | |  |  |
| BDI-I, *n*=999 | 13.08(8.44) | 21.32(10.76) | -13.19^b^ | 1.04e-36 | 9.809^d^ | |  |  |
| BDI-II, *n*=105 | 11.36(8.47) | 15.31(9.79) | -2.00^b^ | 0.049 | 9.399^d^ | |  |  |
| SPIN, *n*=656 | 38.09(11.87) | 41.49(11.41) | -3.31^b^ | 0.001 | 11.535^d^ | |  |  |
| LSAS, *n*=363 | 69.08(22.89) | 73.97(22.93) | -1.92^b^ | 0.056 | 22.906^d^ | |  |  |
| *Notes.* Depending on the scale level we present different statistical values and different measures for effect sizes. ^a^ *X²*-value, ^b^ *t*-value, ^c^ Cramer’s *V*, ^d^ Cohen’s *d*. *Abbreviations.* SAD, social anxiety disorder; SAD+MDD, social anxiety disorder with comorbid major depressive disorder; BDI-I, Beck Depression Inventory version I; BDI-II, Beck Depression Inventory version II; SPIN, Social Phobia Inventory; LSAS, Liebowitz Social Anxiety Scale. | | | | | |  | |  |

## Table S3

| *Descriptive statistics and Levene’s test results.* | | | | | | | | |
| --- | --- | --- | --- | --- | --- | --- | --- | --- |
| PRS | *p*-threshold | Mean  (Controls) | SD  (Controls) | Mean (SAD) | SD (SAD) | Levene *F* | Levene *p* |  |
| Depression | P5e-08 | -0.013 | 1.007 | 0.038 | 0.979 | 0.963 | 3,26e-01 |  |
| Depression | P1e-06 | -0.015 | 1.006 | 0.044 | 0.983 | 0.603 | 4,38e-01 |  |
| Depression | P0.0001 | -0.026 | 0.995 | 0.076 | 1.010 | 0.141 | 7,07e-01 |  |
| Depression | P0.05 | -0.028 | 0.991 | 0.079 | 1.023 | 1.697 | 1,93e-01 |  |
| Depression | P0.5 | -0.030 | 0.988 | 0.085 | 1.029 | 0.794 | 3,73e-01 |  |
| Depression | P1 | -0.030 | 0.987 | 0.084 | 1.032 | 1.339 | 2,47e-01 |  |
| MDD | P5e-08 | -0.018 | 1.017 | 0.052 | 0.946 | 12.319 | 4,53e-04* |  |
| MDD | P1e-06 | -0.021 | 1.018 | 0.063 | 0.945 | 8.743 | 3,00e-03* |  |
| MDD | P0.0001 | -0.022 | 1.018 | 0.062 | 0.948 | 4.610 | 3,20e-02* |  |
| MDD | P0.05 | -0.019 | 1.015 | 0.053 | 0.956 | 5.399 | 2,00e-02* |  |
| MDD | P0.5 | -0.016 | 0.999 | 0.049 | 1.001 | 0.106 | 7,44e-01 |  |
| MDD | P1 | -0.019 | 0.992 | 0.058 | 1.023 | 0.230 | 6,31e-01 |  |
| SCZ | P5e-08 | -0.028 | 1.020 | 0.079 | 0.935 | 13.984 | 1,87e-04* |  |
| SCZ | P1e-06 | -0.033 | 1.022 | 0.095 | 0.930 | 12.304 | 4,56e-04* |  |
| SCZ | P0.0001 | -0.042 | 1.026 | 0.121 | 0.911 | 16.415 | 5,17e-05* |  |
| SCZ | P0.05 | -0.047 | 1.005 | 0.136 | 0.972 | 0.617 | 4,32e-01 |  |
| SCZ | P0.5 | -0.039 | 1.008 | 0.111 | 0.970 | 1.554 | 2,13e-01 |  |
| SCZ | P1 | -0.039 | 1.008 | 0.113 | 0.968 | 1.806 | 1,79e-01 |  |
| BD | P5e-08 | -0.027 | 1.014 | 0.077 | 0.956 | 4.645 | 3,10e-02* |  |
| BD | P1e-06 | -0.025 | 1.016 | 0.073 | 0.950 | 6.183 | 1,30e-02* |  |
| Continues on the next page | | | | | | | | |
| Table S3 continued | | | | | |  |  |  |
| PRS | *p*-threshold | Mean  (Controls) | SD  (Controls) | Mean (SAD) | SD (SAD) | Levene *F* | Levene *p* |  |
| BD | P0.0001 | -0.038 | 1.003 | 0.107 | 0.985 | 0.147 | 7,01e-01 |  |
| BD | P0.05 | -0.051 | 0.995 | 0.144 | 1.002 | 0.011 | 9,18e-01 |  |
| BD | P0.5 | -0.049 | 0.991 | 0.141 | 1.011 | 0.207 | 6,49e-01 |  |
| BD | P1 | -0.049 | 0.990 | 0.140 | 1.015 | 0.492 | 4,83e-01 |  |
| ADHD | P5e-08 | 0.014 | 1.001 | -0.041 | 0.996 | 0.007 | 9,35e-01 |  |
| ADHD | P1e-06 | 0.001 | 1.005 | -0.002 | 0.985 | 0.434 | 5,10e-01 |  |
| ADHD | P0.0001 | 0.013 | 0.995 | -0.038 | 1.012 | 0.106 | 7,44e-01 |  |
| ADHD | P0.05 | 0.013 | 0.997 | -0.036 | 1.009 | 0.439 | 5,07e-01 |  |
| ADHD | P0.5 | 0.008 | 0.991 | -0.022 | 1.027 | 3.968 | 4,60e-02* |  |
| ADHD | P1 | 0.007 | 0.991 | -0.020 | 1.026 | 3.292 | 7,00e-02 |  |
| AN | P5e-08 | -0.027 | 0.974 | 0.078 | 1.068 | 19.529 | 1,01e-05* |  |
| AN | P1e-06 | -0.030 | 0.976 | 0.085 | 1.060 | 13.263 | 2,74e-04* |  |
| AN | P0.0001 | -0.028 | 0.987 | 0.080 | 1.033 | 3.445 | 6,40e-02 |  |
| AN | P0.05 | -0.062 | 1.003 | 0.178 | 0.970 | 2.355 | 1,25e-01 |  |
| AN | P0.5 | -0.061 | 1.010 | 0.174 | 0.951 | 4.869 | 2,70e-02* |  |
| AN | P1 | -0.060 | 1.010 | 0.172 | 0.951 | 4.181 | 4,10e-02* |  |
| ASD | P5e-08 | 0.001 | 1.011 | -0.002 | 0.970 | 3.105 | 7,80e-02 |  |
| ASD | P1e-06 | -0.003 | 0.995 | 0.008 | 1.015 | 1.623 | 2,03e-01 |  |
| ASD | P0.0001 | 0.002 | 1.002 | -0.006 | 0.994 | 0.004 | 9,52e-01 |  |
| ASD | P0.05 | -0.029 | 0.999 | 0.084 | 0.998 | 0.249 | 6,18e-01 |  |
| ASD | P0.5 | -0.022 | 0.990 | 0.063 | 1.027 | 2.516 | 1,13e-01 |  |
| Continues on the next page | | | | | | | | |
| **Table S3** **continued** | | | | | |  |  |  |
| PRS | *p*-threshold | Mean  (Controls) | SD  (Controls) | Mean (SAD) | SD (SAD) | Levene *F* | Levene *p* |  |
| ASD | P1 | -0.021 | 0.989 | 0.061 | 1.029 | 2.904 | 8,80e-02 |  |
| AD | P5e-08 | 0.001 | 1.002 | -0.003 | 0.994 | 0.754 | 9,20e-01 |  |
| AD | P0.0001 | 0.002 | 1.000 | -0.005 | 1.001 | 0.104 | 3,91e-01 |  |
| AD | P0.05 | 0.003 | 0.999 | -0.007 | 1.005 | 0.084 | 1,82e-01 |  |
| AD | P0.5 | 0.001 | 1.003 | -0.002 | 0.990 | 0.182 | 8,96e-01 |  |
| AD | P1 | 0.001 | 1.004 | -0.002 | 0.990 | 0.488 | 8,53e-01 |  |
| *Notes.* The table presents the mean and standard deviation of polygenic risk scores for individuals with social anxiety disorder and controls as well as Levene’s test results. The assumption of homogeneity of variances was violated in 14 tests (indicated using an asterisk *) and **Welch’s ANOVA** was therefore conducted. *Abbreviations.* SD, standard deviation; PRS, polygenic risk score; **ANOVA,** Welch’s Analysis of Variance; Depression, broader definition of depressive symptoms; MDD, major depressive disorder; ANX, anxiety disorder; SCZ, schizophrenia; BD, bipolar disorder; ADHD, attention-deficit//hyperactivity disorder; AN, anorexia nervosa; ASD, autism spectrum disorder; AD, alcohol dependence. | | | | | | | | |

## Table S4

| *Results of the Welch’s Analysis of Variance.* | | | | | |
| --- | --- | --- | --- | --- | --- |
| PRS | *p*- threshold | Welch's *F* | *p*-value | *p_BH_*-adjusted |  |
| AN | P0.5 | 52.457 | 6.05e-13 | 8.47e-12* |  |
| AN | P1 | 51.202 | 1.13e-12 | 7.91e-12* |  |
| SCZ | P0.0001 | 26.530 | 2.81e-07 | 1.31e-06* |  |
| SCZ | P1e-06 | 16.071 | 6.30e-05 | 2.20e-04* |  |
| SCZ | P5e-08 | 11.045 | 9,04e-04 | 2,53e-03* |  |
| AN | P1e-06 | 10.887 | 9,86e-04 | 2,30e-03* |  |
| BD | P5e-08 | 10.043 | 1,55e-03 | 3,10e-03* |  |
| AN | P5e-08 | 9.098 | 2,59e-03 | 4,54e-03* |  |
| BD | P1e-06 | 9.062 | 2,64e-03 | 4,11e-03* |  |
| MDD | P1e-06 | 6.929 | 8,54e-03 | 1,20e-02* |  |
| MDD | P0.0001 | 6.680 | 9,82e-03 | 1,25e-02* |  |
| MDD | P0.05 | 4.835 | 2,80e-02 | 3,27e-02* |  |
| MDD | P5e-08 | 4.712 | 3,01e-02 | 3,24e-02* |  |
| ADHD | P0.5 | 0.750 | 3,87e-01 | 3,87e-01 |  |

*Notes.* ^*^ The asterisk denotes significance at *p_BH_*<0.05 corrected for multiple testing (14 tests) according to Benjamini-Hochberg using Welch’s Analysis of Variance. *Abbreviations.* BH, Benjamini-Hochberg; PRS, polygenic risk score.

## Table S5

*Results of the association analyses between PRS and SAD subphenotypes.*

| PRS | *p*-threshold | BDI-I |  |  | BDI-II |  |  | LSAS |  |  | SPIN |  |  | |
| --- | --- | --- | --- | --- | --- | --- | --- | --- | --- | --- | --- | --- | --- | --- |
|  |  | *r* | *p*-value | *p_BH_*-adjusted | *r* | *p*-value | *p_BH_*-adjusted | *r* | *p*-value | *p_BH_*-adjusted | *r* | *p*-value | *p_BH_*-adjusted | |
| Depression | P0.0001 | 0.038 | 0.222 | 0.444 | 0.048 | 0.637 | 1.000 | 0.037 | 0.481 | 0.905 | 0.006 | 0.877 | 1.000 | |
| Depression | P0.05 | 0.097 | 0.002 | 0.064 | 0.095 | 0.350 | 1.000 | 0.044 | 0.402 | 1.000 | 0.039 | 0.320 | 0.931 | |
| Depression | P0.5 | 0.072 | 0.022 | 0.143 | 0.128 | 0.208 | 0.832 | 0.005 | 0.927 | 1.000 | 0.020 | 0.611 | 1.000 | |
| Depression | P1 | 0.068 | 0.031 | 0.163 | 0.134 | 0.188 | 1.000 | 4.5e-04 | 0.993 | 0.993 | 0.020 | 0.615 | 0.984 | |
| MDD | P5e-08 | 0.049 | 0.117 | 0.374 | 0.021 | 0.839 | 1.000 | 0.041 | 0.443 | 1.000 | 0.002 | 0.950 | 0.981 | |
| MDD | P1e-06 | 0.057 | 0.069 | 0.246 | -0.040 | 0.697 | 1.000 | 0.023 | 0.662 | 1.000 | 0.032 | 0.418 | 0.955 | |
| MDD | P0.0001 | 0.041 | 0.188 | 0.401 | 0.035 | 0.731 | 1.000 | 0.005 | 0.928 | 1.000 | 0.052 | 0.187 | 0.856 | |
| MDD | P1 | 0.049 | 0.121 | 0.322 | 3.1e-05 | 1.000 | 1.000 | -0.021 | 0.692 | 1.000 | -0.003 | 0.937 | 0.999 | |
| ANX | P0.05 | 0.034 | 0.274 | 0.461 | -0.041 | 0.684 | 1.000 | 0.051 | 0.333 | 1.000 | -0.022 | 0.567 | 1.000 | |
| Continues on the next page | | | | | | | | | | | | | |  |
| **Table S5 continued** | | | | | | | | | | | | | |  |
| PRS | *p*-threshold | BDI-I |  |  | BDI-II |  |  | LSAS |  |  | SPIN |  |  | |
|  |  | *r* | *p*-value | *p_BH_*-adjusted | *r* | *p*-value | *p_BH_*-adjusted | *r* | *p*-value | *p_BH_*-adjusted | *r* | *p*-value | *p_BH_*-adjusted | |
| ANX | P0.5 | 0.031 | 0.319 | 0.511 | 0.076 | 0.457 | 1.000 | 0.043 | 0.417 | 1.000 | -0.018 | 0.644 | 0.896 | |
| ANX | P1 | 0.030 | 0.335 | 0.487 | 0.050 | 0.625 | 1.000 | 0.039 | 0.457 | 0.975 | -0.018 | 0.655 | 0.874 | |
| SCZ | P5e-08 | 0.049 | 0.118 | 0.343 | -0.043 | 0.671 | 1.000 | 0.063 | 0.232 | 1.000 | 0.019 | 0.623 | 0.949 | |
| SCZ | P1e-06 | 0.058 | 0.063 | 0.252 | -0.008 | 0.939 | 1.000 | 0.064 | 0.227 | 1.000 | 0.017 | 0.666 | 0.853 | |
| SCZ | P0.0001 | 0.063 | 0.045 | 0.206 | 0.058 | 0.568 | 1.000 | 0.058 | 0.274 | 1.000 | 0.005 | 0.906 | 1.000 | |
| SCZ | P0.05 | 0.004 | 0.901 | 0.962 | 0.130 | 0.200 | 0.913 | 0.113 | 0.034 | 0.358 | 0.003 | 0.934 | 1.000 | |
| SCZ | P0.5 | 0.014 | 0.650 | 0.743 | 0.147 | 0.146 | 1.000 | 0.126 | 0.018 | 0.562 | 0.020 | 0.615 | 1.000 | |
| SCZ | P1 | 0.016 | 0.618 | 0.732 | 0.145 | 0.152 | 1.000 | 0.124 | 0.019 | 0.302 | 0.016 | 0.681 | 0.838 | |
| BD | P5e-08 | 0.047 | 0.131 | 0.322 | -0.038 | 0.711 | 1.000 | 0.082 | 0.121 | 0.777 | 0.023 | 0.564 | 1.000 | |
| BD | P1e-06 | 0.038 | 0.228 | 0.429 | -7.9e-05 | 0.999 | 1.000 | 0.086 | 0.104 | 0.830 | 0.022 | 0.581 | 1.000 | |
| Continues on the next page | | | | | | | | | | | | | |  |

| **Table S5 continued** | | | | | | | | | | | | | |  |
| --- | --- | --- | --- | --- | --- | --- | --- | --- | --- | --- | --- | --- | --- | --- |
| PRS | *p*-threshold | BDI-I |  |  | BDI-II |  |  | LSAS |  |  | SPIN |  |  | |
|  |  | *r* | *p*-value | *p_BH_*-adjusted | *r* | *p*-value | *p_BH_*-adjusted | *r* | *p*-value | *p_BH_*-adjusted | *r* | *p*-value | *p_BH_*-adjusted | |
| BD | P0.0001 | 0.001 | 0.987 | 1.000 | 0.009 | 0.933 | 1.000 | 0.037 | 0.480 | 0.961 | 0.037 | 0.344 | 0.917 | |
| BD | P0.05 | 0.010 | 0.751 | 0.829 | -0.018 | 0.861 | 1.000 | -0.017 | 0.756 | 0.968 | 0.048 | 0.220 | 0.880 | |
| BD | P0.5 | 0.031 | 0.330 | 0.503 | -0.002 | 0.983 | 1.000 | -0.042 | 0.427 | 1.000 | 0.061 | 0.118 | 1.000 | |
| BD | P1 | 0.030 | 0.341 | 0.474 | 0.005 | 0.960 | 1.000 | -0.047 | 0.374 | 1.000 | 0.064 | 0.103 | 1.000 | |
| AN | P5e-08 | -0.026 | 0.414 | 0.552 | 0.136 | 0.178 | 1.000 | 0.023 | 0.659 | 1.000 | -2.0e-04 | 0.996 | 0.996 | |
| AN | P1e-06 | 4.9e-04 | 0.988 | 0.988 | 0.158 | 0.119 | 1.000 | -0.005 | 0.930 | 0.992 | 0.019 | 0.636 | 0.926 | |
| AN | P0.0001 | 0.026 | 0.415 | 0.531 | 0.196 | 0.052 | 1.000 | -0.008 | 0.885 | 1.000 | 0.055 | 0.158 | 1.000 | |
| AN | P0.05 | -0.025 | 0.427 | 0.525 | 0.023 | 0.820 | 1.000 | 0.014 | 0.785 | 0.967 | -0.036 | 0.359 | 0.884 | |
| AN | P0.5 | -0.042 | 0.184 | 0.421 | 0.078 | 0.444 | 1.000 | -0.026 | 0.629 | 1.000 | -0.042 | 0.281 | 0.999 | |
| AN | P1 | -0.037 | 0.242 | 0.429 | 0.070 | 0.493 | 1.000 | -0.024 | 0.654 | 1.000 | -0.041 | 0.298 | 0.954 | |
| Continues on the next page | | | | | | | | | | | | | |  |
| **Table S5 continued** | | | | | | | | | | | | | |  |
| PRS | *p*-threshold | BDI-I |  |  | BDI-II |  |  | LSAS |  |  | SPIN |  |  | |
|  |  | *r* | *p*-value | *p_BH_*-adjusted | *r* | *p*-value | *p_BH_*-adjusted | *r* | *p*-value | *p_BH_*-adjusted | *r* | *p*-value | *p_BH_*-adjusted | |
| ASD | P0.05 | 0.072 | 0.021 | 0.168 | 0.052 | 0.606 | 1.000 | -0.002 | 0.963 | 0.994 | 0.080 | 0.042 | 1.000 | |
| ASD | P0.5 | 0.078 | 0.013 | 0.216 | 0.028 | 0.784 | 1.000 | -0.019 | 0.718 | 0.957 | 0.055 | 0.164 | 1.000 | |
| ASD | P1 | 0.073 | 0.020 | 0.218 | 0.012 | 0.903 | 1.000 | -0.019 | 0.714 | 0.993 | 0.054 | 0.168 | 0.896 | |

*Notes.* Results were corrected for multiple testing (32 tests) using the family-wise error adjusted *p*-value according to Benjamini-Hochberg.

*Abbreviations.* PRS, polygenic risk score; Depression, broader definition of depressive symptoms; MDD, major depressive disorder; ANX, anxiety disorder; SCZ, schizophrenia; BD, bipolar disorder; AN, anorexia nervosa; ASD, autism spectrum disorder; BDI-I, Beck Depression Inventory version I; BDI-II, Beck Depression Inventory version II; SPIN, Social Phobia Inventory; LSAS, Liebowitz Social Anxiety Scale.

## Table S6

*Results of the PRS comparing SAD and SAD+MDD.*

| PRS | *p*-threshold | *F*-value | *p*-value | *p_BH_*-adjusted |
| --- | --- | --- | --- | --- |
| Depression | P0.0001 | 0.032 | 0.858 | 1.000 |
| Depression | P0.05 | 1.628 | 0.202 | 0.719 |
| Depression | P0.5 | 1.280 | 0.258 | 0.636 |
| Depression | P1 | 1.335 | 0.248 | 0.662 |
| MDD | P5e08 | 2.131 | 0.145 | 0.579 |
| MDD | P1e06 | 3.442 | 0.064 | 0.511 |
| MDD | P0.0001 | 1.174 | 0.279 | 0.637 |
| MDD | P1 | 0.147 | 0.701 | 1.000 |
| ANX | P0.05 | 0.053 | 0.818 | 1.000 |
| ANX | P0.5 | 0.000 | 0.997 | 1.000 |
| ANX | P1 | 0.001 | 0.972 | 1.000 |
| SCZ | P5e08 | 0.991 | 0.320 | 0.682 |
| SCZ | P1e06 | 0.815 | 0.367 | 0.734 |
| SCZ | P0.0001 | 2.679 | 0.102 | 0.544 |
| SCZ | P0.05 | 0.041 | 0.840 | 1.000 |
| SCZ | P0.5 | 0.061 | 0.805 | 1.000 |
| SCZ | P1 | 0.116 | 0.734 | 1.000 |
| BD | P5e08 | 3.485 | 0.062 | 0.663 |
| BD | P1e06 | 3.068 | 0.080 | 0.513 |
| BD | P0.0001 | 0.637 | 0.425 | 0.756 |
| Continues on the next page | | | | |
| Table S6 continued | | | | |
| PGS | *p*-threshold | *F*-value | *p*-value | *p_BH_*-adjusted |
| BD | P0.05 | 0.245 | 0.621 | 1.000 |
| BD | P0.5 | 8.0e-07 | 0.999 | 0.999 |
| BD | P1 | 9.8e-05 | 0.992 | 1.000 |
| AN | P5e08 | 8.691 | 0.003 | 0.104 |
| AN | P1e06 | 6.634 | 0.010 | 0.162 |
| AN | P0.0001 | 2.675 | 0.102 | 0.467 |
| AN | P0.05 | 0.004 | 0.947 | 1.000 |
| AN | P0.5 | 1.0e-04 | 0.992 | 1.000 |
| AN | P1 | 8.4e-05 | 0.993 | 1.000 |
| ASD | P0.05 | 0.807 | 0.369 | 0.695 |
| ASD | P0.5 | 1.567 | 0.211 | 0.614 |
| ASD | P1 | 1.611 | 0.205 | 0.655 |

*Notes.* We corrected for multiple testing using the family-wise error adjusted *p*-value according to Benjamini-Hochberg (range 0-1). *Abbreviations.* PRS, polygenic risk score; Depression, broader definition of depressive symptoms; MDD, major depressive disorder; ANX, anxiety disorder; SCZ, schizophrenia; BD, bipolar disorder; AN, anorexia nervosa; ASD, autism spectrum disorder.
